# Achieving Robust Compressive Sensing Seismic Acquisition with a Two-Step Sampling Approach †

**DOI:** 10.3390/s23239519

**Published:** 2023-11-30

**Authors:** Anna Titova, Michael B. Wakin, Ali C. Tura

**Affiliations:** 1 Department of Geophysics, Colorado School of Mines, 1500 Illinois St., Golden, CO 80401, USA; alitura@mines.edu; 2 Electrical Engineering Department, Colorado School of Mines, 1500 Illinois St., Golden, CO 80401, USA; mwakin@mines.edu

**Keywords:** signal sampling, survey design, compressive sensing, seismic acquisition

## Abstract

The compressive sensing (CS) framework offers a cost-effective alternative to dense alias-free sampling. Designing seismic layouts based on the CS technique imposes the use of specific sampling patterns in addition to the logistical and geophysical requirements. We propose a two-step design process for generating CS-based schemes suitable for seismic applications. During the first step, uniform random sampling is used to generate a random scheme, which is supported theoretically by the restricted isometry property. Following that, designated samples are added to the random scheme to control the maximum distance between adjacent sources (or receivers). The null space property theoretically justifies the additional samples of the second step. Our sampling method generates sampling patterns with a CS theoretical background, controlled distance between adjacent samples, and a flexible number of active and omitted samples. The robustness of two-step sampling schemes for reallocated samples is investigated and CS reconstruction tests are performed. In addition, using this approach, a CS-based 3D seismic survey is designed, and the distributions of traces in fold maps and rose diagrams are analyzed. It is shown that the two-step scheme is suitable for CS-based seismic surveys and field applications.

## 1. Introduction

Seismic surveys are designed to balance cost, logistics, and a spatial sampling interval, which is crucial for recording a seismic wavefield. The proper (alias-free) spatial sampling of the seismic wavefield is achieved when the distance between adjacent sources (or receivers) Δd does not exceed the Nyquist sampling criterion $\Delta d_{Nyq}$: $\Delta d\leq \Delta d_{Nyq}=\frac{V_{min}}{2f_{max}}$, where $V_{min}$ is the minimum velocity of the media and $f_{max}$ is the maximum frequency of the source [1]. Depending on $V_{min}$ and $f_{max}$, the Nyquist criterion may be very small, e.g., 1 m. The sampling theorem can be expanded to relax the Nyquist criterion, but these expansions require measurements of the wavefield gradient [2,3]. Even so, the successful application of the sampling theorem and its expansions may be challenging when the knowledge of $V_{min}$ over the area of interest is limited.

The alias-free sampling of a seismic wavefield in all spatial directions is rarely satisfied in practice due to the cost constraints of the survey. Various solutions have been adopted and developed to overcome the issue of spatial aliasing. For instance, in typical exploration survey layouts, at least one or two out of four spatial coordinates are densely sampled to facilitate the proper removal of unwanted linear noise [4]. This type of noise is usually represented by the surface waves that are suffering the most from the sparse aliasing sampling intervals. On the data processing side, different algorithms can be applied to mitigate the aliasing in the data, for instance, reconstruction methods based on the prediction error filter [5,6]. In addition to a long-standing dilemma of shooting a dense survey or working with aliased seismic gathers, the ideal regular seismic layout can be compromised by field deployment circumstances: obstacles and areas without access, equipment malfunction, zones with poor recording conditions, logistical constraints, and surface topography. These long-standing, common, and persistent seismic acquisition challenges motivate us to consider non-regular and non-dense survey layouts.

Recent advances in signal processing have paved the way for a new cost-effective acquisition paradigm called compressive sensing (CS) [7]. The foundation of CS is the concept of signal sparsity, which is when almost all the energy of a signal is contained in a few transform coefficients of the signal. The key elements of the CS framework include the sampling scheme, sparse transform, and the reconstruction algorithm. Together, they form a framework that can solve undetermined problems, especially those that are common in seismic data processing. One of the earliest works on the application of compressive sensing for seismic signals was published by [8]. The field implementation of CS-based acquisition has been pioneered by [9] and further practiced by [10]. It is worth pointing out that, even before the first applications of the CS framework for the problems of seismic data interpolation, a vast amount of techniques for data reconstruction and dealiasing of spatial wavenumbers has been developed [6]. These techniques are being continuously improved [11]. The CS framework stands out from these methods because it constrains in a direct manner the type of sampling pattern needed for the CS reconstruction workflow to succeed.

We focus on CS-based sampling schemes and the application of these schemes for seismic CS. Different CS-based sampling methods can be found in the literature. However, the authors of these methods do not assess the robustness of the prepared CS-based schemes to sample perturbations or reallocations. The latter is an essential consideration for seismic CS-based design since the prepared layout of sources and receivers can be disturbed during the field deployment. In addition, only a few of the proposed CS-based sampling methods are supplemented with complete instructions for generating a proposed sampling scheme.

We organize our study as follows. Firstly, we formulate the criteria for CS-based sampling schemes and analyze the strengths and weaknesses of the available sampling methods. Secondly, guided by the theory of the CS framework, we introduce and implement our two-step sampling approach. We evaluate the performance of two-step sampling schemes through CS reconstruction and compare them with the results obtained for the jittered schemes. We also compare the sensitivity of two-step and jittered sampling schemes for sample reallocations. This comparison demonstrates that the two-step scheme is more robust in the face of perturbations in the sampling scheme.

A mathematical formulation of the CS framework for seismic applications is provided in Appendix A. Furthermore, in Appendix B, we summarize different tools that can be used to analyze the CS-based schemes.

## 2. Overview of Sampling Methods

Important characteristics of CS-based sampling schemes include the following: CS theoretical guarantees, tolerance to perturbations of scheme samples, and flexibility to incorporate geophysical parameters and logistical constraints. Many methods have been proposed for generating CS-based sampling schemes for seismic applications. We briefly describe these methods by clustering them into five main groups based on sampling mechanisms.

The first group of methods includes schemes with random source (receiver) spacing. The random samples may be generated by taking *M* samples out of *N* uniformly at random (M<N). Consequently, the maximum distance between adjacent samples in such schemes is not constrained and can vary in a wide range. For sparse signals in the Fourier domain, the restricted isometry property guarantees successful CS reconstruction with random samples [12]. However, for sparse signals under a transform with localized basis functions (e.g., curvelet transform), the large distance between adjacent sources (or receivers) deteriorates the CS reconstruction results [13]. Therefore, the random sampling schemes are not recommended due to implied limitations on the sparse transforms. In addition to that consideration, the maximum distance between adjacent sources (or receivers) is constrained by the size of the Fresnel zone: the Fresnel zone should be adequately sampled to ensure that CS-reconstructed data represent the subsurface of interest [14,15,16].

The second group includes methods that generate sampling schemes by solving an optimization problem. In most of these methods, the objective functions are related to the Fourier spectrum of a sampling scheme: a maximum non-DC component of the spectrum, also known as coherence [17], a weighted sum of the non-DC amplitudes of the spectrum [18], an average coherence [19], and a coherence map [20]. These methods minimize (or maximize) the objective function, assuming that the largest distance between adjacent sources (or receivers) is fixed and relatively small. The performance of these methods is not discussed when the obstructions limit access to a survey area, and consequently, the largest distance assumption is violated. Also, the coherence does lend itself to CS theoretical guarantees [12], but the assumptions for these guarantees rarely hold in practice. Moreover, the proposition that sampling schemes with smaller coherence promote better reconstruction results is uncertain since some studies support this proposition [21,22,23,24,25] and others disprove it [26,27,28,29,30,31]. Finally, coherence computation includes a sparse transform (e.g., the Fourier transform), making these methods computationally expensive for large-scale 3D surveys.

The third group uses the difference sets from combinatorial number theory to define CS-based schemes [24,31,32]. A difference set with parameters (α,β,γ) contains β elements taken from the bigger set {1,2,…,α}, α>β, such that each element of the set {1,2,…,α} can be written as the difference of two elements of this set in exactly γ ways. The difference sets may be excellent candidates for CS-based schemes from a mathematical point of view. For example, these sampling schemes achieve the smallest coherence when the Fourier transform is taken as the sparse transform. In addition, all non-DC amplitudes of the Fourier spectrum of a difference set are equal. The disadvantages of using the difference set for seismic survey design start with the absence of control of the distances between adjacent sources (or receivers). Second, the difference set limits the survey parameters, such as the number of sources (or receivers) used in the field, because the difference sets exist only for specific combinations of parameters α, β, and γ. Finally, the difference sets are sensitive to any changes in the scheme: the smallest coherence may no longer be achieved with a few reallocated samples. Such sampling schemes impose additional restrictions on the field implementations of CS-based surveys.

The methods in the fourth group are based on a sub-interval strategy [8,33,34,35]. Implementations of this strategy include (i) splitting a source (or receiver) line into sub-intervals and (ii) taking samples within each sub-interval based on a probability distribution. This strategy is devised to control the distance between adjacent sources (or receivers). A recent study by [36] can be used to theoretically investigate the CS reconstruction performance of these methods. One of the disadvantages of sub-interval methods is that repetitive sampling patterns may appear due to the independent choice of the samples in each sub-interval. In addition, the sub-interval strategy may limit the choice of survey parameters. For example, in the jittered sampling, the number of acquired data points *M* should be a sub-multiple of the number of data points after reconstruction *N* [8]. In other methods from the sub-interval group [33,35], the relationship between *M* and *N* has been made more flexible by randomly varying the number of samples in each subinterval $n_{sub}$ and the length of each subinterval $l_{sub}$. Consequently, two additional parameters are introduced in the sampling scheme generation process. The relationship between these four parameters (*M*, *N*, $n_{sub}$, and $l_{sub}$) has to be analyzed to understand how to generate a sampling scheme once specific *M* and *N* are given.

Low-discrepancy sequences define the fifth group of sampling approaches for building CS-based schemes. In seismic exploration, these sequences were first applied as an optimum discretization scheme for migration algorithms. In particular, ref. [37] used the Halton and Hammersley points to provide a near-optimal discretization for the prestack Kirchhoff migration. As for the CS application, the low-discrepancy sequences may be good candidates because these sequences avoid clusters of sources (or receivers) and large distances between adjacent sources (or receivers). On the other hand, the disadvantages of these sequences include (i) the absence of known CS recovery guarantees and (ii) a logistically challenging implementation of these schemes in the field due to off-grid locations of sources (or receivers). Furthermore, the numerical studies on CS reconstruction with the Hammersley points demonstrate discouraging results [38] and, finally, low-discrepancy sequences may require additional transformation to reduce aliasing [39].

The two-step sampling method was initially proposed by [40]. This paper describes this method in detail and performs different numerical experiments with the two-step sampling schemes. The two-step sampling approach provides sampling schemes with the CS theoretical background, the controlled distance between adjacent sources (or receivers), and the flexible choice of seismic survey parameters. The method combines two types of sampling: random and designated. Random samples are needed to achieve a CS theoretically compliant sampling. The designated samples are purposefully added to the random scheme to control the maximum distance between adjacent sources (or receivers). The random and designated samples are generated sequentially, one after the other. Two-step sampling can be applied in the presence of obstructions in the field and does not limit the choice of seismic survey parameters. In addition, our approach generates schemes that are robust to modifications in a prepared layout of sources (or receivers). The theoretical considerations for the two-step sampling are built based on the Fourier basis. However, the use of this sampling method is not limited to the Fourier sparse transform. In fact, the numerical CS reconstruction experiments are performed with the curvelet sparse transform.

## 3. Two-Step Sampling Mechanism

### 3.1. Theoretical Background

Two-step sampling merges random and designated samples. The random samples are needed to obtain CS recovery guarantees for the sensing matrix A when the Fourier transform is used as the sparse transform. In this case, the sensing matrix is a member of a special class of matrices—the random partial Fourier matrices. These matrices are built by selecting a subset of *M* rows uniformly at random from the *N* rows of the Fourier matrix (M<N). It is known that this class of matrices satisfies the restricted isometry property (RIP) with high probability [12]. Namely, the random partial Fourier matrices with the number of measurements *M* satisfying the following inequality

(1) $$M\leq cs\ln^{4}N,$$

have the RIP of order *s* with the arbitrary constant c>0.

The random sampling scheme does not control the distance between adjacent samples, and consequently, the CS reconstruction of signals that are not perfectly sparse may deteriorate. Also, the distance between adjacent samples should be controlled to meet the Fresnel zone requirement of the seismic survey. The designated samples may be added to address this issue. Mathematically, adding designated samples into the random sampling scheme is equivalent to appending new rows into the sensing matrix, which is accomplished by adding new binary rows to the matrix R while keeping the matrix C fixed (Appendix A). These additional rows may change the restricted isometry constant (RIC), and the RIP will no longer hold if the RIC becomes too large, ceasing the CS recovery guarantees.

Adding designated samples into the random sampling scheme and, at the same time, preserving the CS recovery guarantees may be achieved by the $\ell_{2}$ robust null space property (NSP) (Equation (A8)). First, it is known that sensing matrices with $\delta_{2s}<4/\sqrt{41}$ satisfy the $\ell_{2}$-robust NSP of order *s* with 0<ρ<1 and τ>0 depending only on $\delta_{2s}$ [12]. Secondly, we demonstrate in Appendix C that the $\ell_{2}$ robust NSP stays valid with additional rows appended into the sensing matrix. The combination of the RIP and the NSP provides the schemes with theoretical guarantees and, at the same time, a controlled distance between adjacent samples.

### 3.2. Implementation

The implementation of two-step sampling consists of generating a random scheme and inserting designated samples. We introduce the following notation to facilitate an explanation of our method. Let the set $\Omega_{R}$ contain $M_{R}$ random samples obtained during the first step, and let the set $\Omega_{D}$ include $M_{D}$ designated samples associated with the second step. The union of these two sets is the set $\Omega_{A}=\Omega_{R}\cup \Omega_{D}$, which contains all samples active in the field. The cardinality of the set $\Omega_{A}$ is equal to $M_{A}=M_{R}+M_{D}$, or in other words, $\mathrm{card}\left(M_{A}\right)=\mathrm{card}\left(M_{R}\right)+\mathrm{card}\left(M_{D}\right)$. The set $\Omega_{O}$ denotes a set of omitted samples for which field data are not recorded, and the set $\Omega =\Omega_{O}\cup \Omega_{A}$ contains active and omitted samples with $\mathrm{card}\left(\Omega \right)=M_{O}+M_{A}=N$. We define a gap in a sampling scheme as the distance between adjacent active samples. We consider that samples are placed on a grid, and consequently, the gap can be measured in grid steps. For example, the gap size of *g* means there are *g* empty grid steps between adjacent active samples.

**Figure 1:**
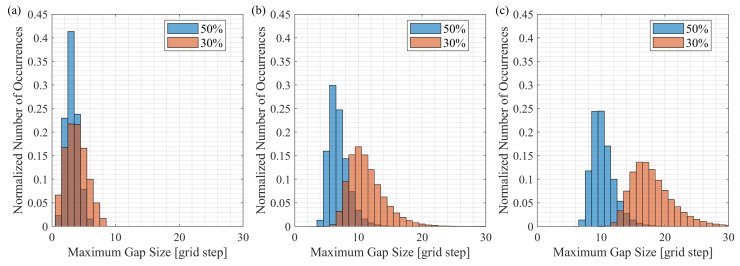
The histograms of the maximum gap sizes in 1,000,000 random schemes with 30% and 50% of active samples for (**a**) N=10, (**b**) N=100, and (**c**) N=1000. With the increase in the total number of samples (active and omitted), the smallest maximum gap size increases.

**Figure 2:**
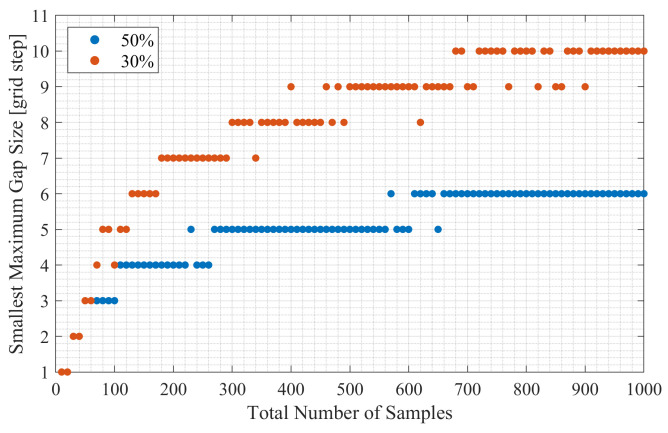
The relationship between the smallest maximum gap size and the total number of samples in random sampling schemes. The maximum gap size exceeds four grid steps once the total number of samples exceeds 100.

Firstly, we study how often a random number generator creates random sampling schemes with a small maximum gap. In this experiment, we first generated random sampling schemes with 30% and 50% of active samples, and then we varied the total number of samples in the schemes (*N*). The histograms of the maximum gap sizes in random schemes with 30% and 50% of active samples are shown in Figure 1a–c for the cases of N=10, N=100, and N=1000, respectively. We used 1,000,000 trials for each case. Figure 1 demonstrates that, as the total number of samples increases, the smallest maximum gap size that appeared over 1,000,000 trials increases. This observation is also supported by Figure 2, which shows the relationship between the smallest maximum gap size in random sampling schemes and the total number of samples. According to Figure 2, when the total number of samples in the scheme exceeds 100, the random sampling schemes with 50% of active samples will have a maximum gap size not smaller than four grid steps. It is worth highlighting that Figure 2 shows the smallest maximum gap size, and the median maximum gap size is larger, as can be observed in Figure 1. Therefore, when the total number of samples is relatively large (e.g., N=500), the small gaps (e.g., gap size of 3) will rarely appear in the schemes with samples chosen uniformly at random.

The designated samples can be added to control the gap size, but at the same time, the total number of samples in the sampling scheme becomes uncontrolled. Analyzing random schemes, one can notice that the gaps in such schemes have different sizes and are distributed differently within the scheme. To achieve the sampling schemes with the fixed number of active samples ($M_{A}=M_{R}+M_{D}$) and predetermined maximum gap size $g_{max}$ at the same time, we formulate the following optimization problem:

(2) $$\begin{array}{c} \Omega_{R}=\arg\max_{\Omega_{R}^{\prime }\subset \left[N\right]}\mathrm{card}\left(\Omega_{R}^{\prime }\right)\;\;\mathrm{subject}\;\mathrm{to}\;\;\\ f\left(\Omega_{A}\right)=g_{max}\;\;\mathrm{and}\;\;\mathrm{card}\left(\Omega_{A}\right)=M_{A}, \end{array}$$

where $f(\Omega_{A})$ is a function that computes the largest gap in the sampling scheme. According to Equation (2), we are interested in finding a realization of random sampling $\Omega_{R}$ with the maximum possible number of random samples $M_{R}$ such that the number of all active samples will not exceed $M_{A}=M_{R}+M_{D}$. The more random samples taken in the first step, the larger the sparsity level *s* of the RIP. Thus, signals with more non-zero transform coefficients may be reconstructed.

Equation (2) outlines three main parameters involved in the search of a random sampling scheme: the maximum gap size $g_{max}$, the number of random samples $M_{R}$, and the number of active samples $M_{A}$. We implemented the following steps to numerically study the space of these three parameters:

(i) Generate a random sampling scheme with ${\tilde{M}}_{R}$ samples;
(ii) Add as many samples as needed to fill in all gaps larger than the gap threshold ${\tilde{g}}_{max}$;
(iii) Count the number of samples ${\tilde{M}}_{A}$ in the resulting scheme.

**Figure 3:**
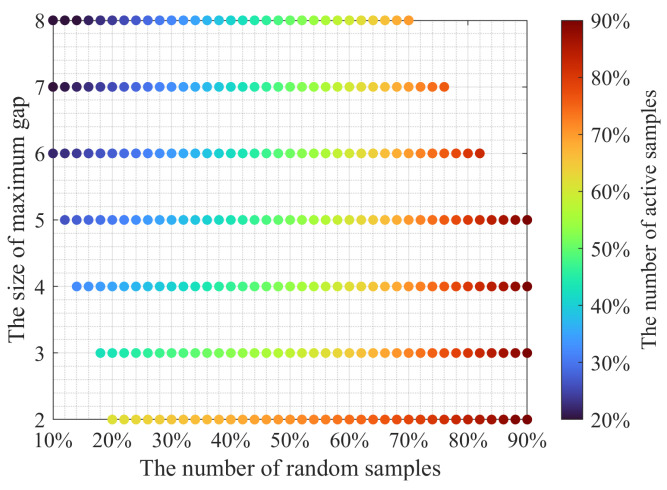
The relationship between three key parameters of two-step sampling: the number of random samples $M_{R}$ taken during the first step, the maximum gap size $g_{max}$ achieved with the designated samples, and the number of active samples $M_{A}$ in the two-step scheme. This empirical relationship shows that, as the threshold on the maximum gap size increases, more random samples are present in the sampling scheme for a fixed number of active samples.

Steps (i)–(iii) are repeated 100 times for each pair ${\tilde{M}}_{R}$ and ${\tilde{g}}_{max}$, where ${\tilde{M}}_{R}/N\in \left[0.1:0.02:0.9\right]$ and ${\tilde{g}}_{max}\in \left[2:1:8\right]$. As for ${\tilde{M}}_{A}$, this parameter is not constrained and may be different for a given pair ${\tilde{M}}_{R}$ and ${\tilde{g}}_{max}$. The median ${\tilde{M}}_{A}^{med}$ of 100 different ${\tilde{M}}_{A}$ is plotted in Figure 3 as a function of ${\tilde{M}}_{R}$ and ${\tilde{g}}_{max}$. Parameters ${\tilde{M}}_{R}$ and ${\tilde{M}}_{A}^{med}$ are normalized by the number of all samples in the scheme *N*, which is equal to 1000 in this particular case. Other values of *N* lead to a very similar relationship between ${\tilde{M}}_{R}$, ${\tilde{g}}_{max}$, and ${\tilde{M}}_{A}^{med}$. The results of studying the parameter space of the two-step sampling approach are shown in Figure 3. At the bottom-left corner of the plot, we observe that a maximum of 30% of random samples may be taken to fill in all gaps larger than three grid steps and not to exceed 50% of active samples. As the gap threshold increases, more random samples can be taken. Finally, with the gap threshold of 8 grid steps, the second step may not be needed because a random scheme with 50% of active samples will likely have the largest gap size of 8 grid steps.

During the second step, the random sampling scheme is scanned to identify positions of the gaps larger than a given threshold. Furthermore, each gap is filled with a minimum number of designated samples until the gap threshold is achieved. We perform the gap-filling process using two options: recursive and jittered. The recursive gap-filling option includes the following steps:

(i) Generate a local random scheme for each identified gap;
(ii) Merge the local random scheme into the scheme from step (i);
(iii) Compute the gaps in the updated scheme;
(iv) Repeat steps (i)–(iii) until all large gaps are filled.

The neighboring samples outside of the selected gaps can be considered to avoid repetitive patterns while filling these gaps in. The jittered gap-filling option uses jittered sampling to fill in the large gaps. Other sampling techniques may be considered during the second step since the NSP does not impose any restrictions on the choice of designated samples.

### 3.3. Fourier Spectrum of Sampling Schemes

Once the sampling scheme is created, its characteristics need to be verified before moving to large-scale experiments. The sampling scheme represents a sequence of ones and zeros. The analysis of such binary sequence cannot be straightforward, especially if the sequence is long, for instance, 1000 samples. For this purpose, we consider the average Fourier spectrum of sampling schemes $f^{ave}$ defined as follows:

(3) $$f^{ave}=\frac{1}{J}\sum_{j=1}^{J}\left|f^{j}\right|,$$

where $f^{j}$ is the Fourier spectrum of the *j*-th sampling scheme, and *j* indexes the realizations of sampling schemes generated by the same method. In the overview of different sampling methods, we noted that there is no consensus about using coherence, which is directly related to the Fourier spectrum of the sampling scheme, as a measure of goodness of the sampling scheme. In this paper, we use the average Fourier spectrum to analyze the statistical properties of these schemes in the Fourier domain. The average Fourier spectrum can also indicate the presence of specific redundant sampling patterns in the sampling schemes.

**Figure 4:**
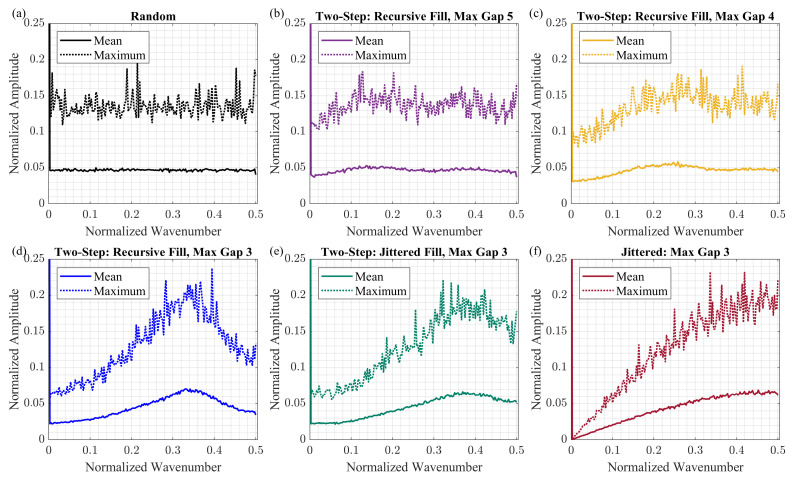
The Fourier spectra analysis for different sampling schemes: (**a**) random, (**b**) two-step with recursive gap-filling option and the maximum gap of 5, (**c**) two-step with recursive gap-filling option and the maximum gap of 4, (**d**) two-step with recursive gap-filling option and the maximum gap of 3, (**e**) two-step with jittered gap-filling option and the maximum gap of 3, and (**f**) jittered with maximum gap of 3. At the zero wavenumber, the value of the spectrum is equal to one. The sampling schemes generated by different methods contain different sampling patterns that are revealed by the average Fourier spectrum.

Figure 4 shows the Fourier spectra computed based on Equation (3) with J=1000 for each sampling method. We consider that each scheme has 180 active samples ($M_{A}=180$) and 180 omitted samples ($M_{O}=180$), and the total number of samples *N* equals 360. We normalized the average Fourier spectrum by the number of active samples.

For the random schemes, the values of the average Fourier spectrum do not change significantly for all non-zero wavenumbers (Figure 4a). In the case of two-step schemes with the maximum gap size of 5 grid steps, about 95% of active samples are random. Consequently, the average spectrum of two-step schemes with $g_{max}=5$ resembles the almost flat average spectrum of the random schemes (Figure 4b). As the number of random samples and the maximum gap size in two-step schemes decreases, the average spectrum changes its shape. For the case of two-step schemes with the maximum gap size of 3 grid steps, the average spectrum achieves its maximum at the normalized wavenumber of about 0.3. We observe that the gap-filling option may also influence the shape of the average spectrum. For instance, the average spectra for two-step schemes are different for recursive and jittered gap-filling options, even though the maximum gap size is the same in both cases (Figure 4d,e). Finally, for the jittered schemes, the values of the average spectrum linearly increase as the normalized wavenumber increases (Figure 4f).

We also report the maximum value of the Fourier spectrum, which was computed as

(4) $$f^{max}=\max_{j=1\cdots J}\left|f^{j}\right|,$$

where $f^{j}$ is the Fourier spectrum of the *j*-th sampling scheme, and *j* indexes the realizations of sampling schemes generated by the same method. As it is shown in Figure 4, the curves corresponding to the maximum follow the shape of the average Fourier spectra.

In the generated two-step schemes, the second-step samples are used to meet the maximum gap size requirement. And even though both gap-filling options, recursive and jittered, rely on random sampling, the second-step samples are placed not irrespectively to the surrounding first-step samples. This characteristic differentiates two-step sampling from uniform random sampling, where all samples are placed uniformly at random.

## 4. Numerical Experiments

### 4.1. Generating and Modifying Sampling Schemes

In this section, we describe the sampling schemes further analyzed with the average Fourier spectrum, CS reconstruction tests, fold maps, and rose diagrams of an orthogonal survey. We define the initial sampling schemes as those generated by a sampling method without applying additional subsequent changes. Three types of initial sampling schemes are considered. One of them is generated by the jittered sampling method. The two-step approach with recursive and jittered gap-filling options generates two other types. Each scheme has 360 samples (N=360), and 180 out of 360 samples are active ($M_{A}=180$). Thus, other 180 samples are omitted ($M_{O}=N-M_{A}=180$), which implies a decimation ratio of 50%. The maximum gap size in these schemes equals 3 grid steps.

In our experiments, we are also interested in evaluating the performance of different sampling schemes whose several initial sample locations have been changed. We define the modified sampling schemes as those generated by a sampling method and subsequently modified by a specific algorithm. Studying the modified sampling schemes is particularly important for seismic CS applications because unforeseen physical obstructions in the field may lead to changes in the prepared survey layout. Also, some sources (or receivers) can be classified as unacceptable for processing and removed from the recorded data, introducing additional distortions to the initial acquisition layout.

We constructed the following modification process to imitate distortions that may happen with a sampling scheme during its field deployment:

(i) Identify vacant positions in an initial scheme;
(ii) Modify the initial scheme by moving *K* samples from their old positions to new positions from step (i);
(iii) Repeat the modification process $N_{trials}$ times for each *K* and each scheme to consider different scenarios of new positions.

In this modification algorithm, the new positions of *K* samples are chosen from the vacant positions such that the maximum gap size in the modified scheme remains the same as in the initial scheme. Also, this procedure avoids creating large gaps because it is known that sampling schemes with large gaps are not desirable for seismic surveys.

As the samples are reallocated, the old positions of *K* samples cannot be considered as the new locations for any reallocated sample. This setting intends to imitate obstacles that may block the prepared positions of the sources (receivers) in the field. Every input scheme will have $N_{trials}$ modified versions, and each of them can be stored for further analysis. It is also possible to choose one modified version among all modification trials $N_{trials}$ using a criterion for the sampling scheme characterization.

The modified sampling schemes for our numerical tests have been generated for each of the three considered scheme types: jittered sampling, two-step sampling with recursive gap-filling option, and two-step sampling with jittered gap-filling option. The parameters of modified schemes include the number of all samples *N*, the number of active samples $M_{A}$, the maximum gap size, and the number of modified samples $K<M_{A}$. The first three parameters are taken the same as in the initial sampling schemes described above. In the modification algorithm, $N_{trials}$ was set as 100, and coherence was computed for every modified version. For further analysis, modified schemes with the largest coherence value were selected.

### 4.2. Average Fourier Spectra

In this section, we analyze the influence of reallocated samples in a scheme on the shape of the average Fourier spectra. In the case of the initial sampling schemes, we consider 500 realizations for each type with the parameters described in Section 4.1. The modified schemes are also built with considerations outlined in Section 4.1. We consider two cases of the number of modified samples *K*: a mild number of reallocated samples K=5 and a more substantial number of reallocated samples K=30.

**Figure 5:**
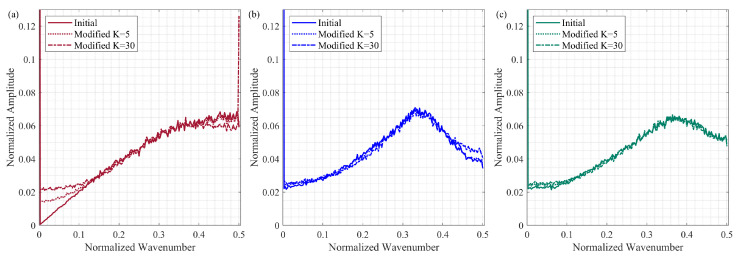
The average Fourier spectra for the initial and modified sampling schemes that are generated according to different sampling methods: (**a**) jittered, (**b**) two-step with the recursive gap-filling option, (**c**) two-step with the jittered gap-filling option. At the zero wavenumber, the value of the spectrum is equal to one. The values of all other components vary in range between 0 and 0.15. For the two-step sampling schemes, the perturbations in the sample locations do not lead to changes in the averaged Fourier spectrum. As for jittered schemes, the sample perturbations change the average Fourier spectrum at high and low wavenumbers.

Figure 5 shows the average Fourier spectra for each group of schemes before and after modifications. We observe that, for two-step schemes, the average spectra are slightly affected by the reallocated samples for both the gap-filling options: recursive (Figure 5b) and jittered (Figure 5c). However, for the jittered schemes, as the number of modified samples increases, deviations from the initial average Fourier spectrum appear for the entire range of the wavenumbers (Figure 5a). Even with a small number of modified samples (K=5), the new jittered schemes are no longer featured by the small values of the average Fourier spectrum at the low wavenumbers (<0.1). As for the case of K=30, the average Fourier spectrum of modified jittered schemes has the spike at the largest normalized wavenumber 0.5. This spike indicates that the modified schemes contain a relatively long aliasing sampling pattern—a portion of a sampling scheme wherein active and omitted samples are regularly alternated. The aliasing sampling patterns occur in the jittered schemes since samples within each sub-interval are chosen independently from neighboring sub-intervals. Although these patterns may be short in the initially populated jittered schemes, several short aliasing patterns may accidentally merge into one long pattern due to the reallocation of the samples.

### 4.3. Reconstruction Test

The reconstruction test aims to assess the performance of the two-step schemes in the CS reconstruction experiments. In these experiments, a seismic signal is decimated in the spatial direction according to different schemes, and no decimation is applied along the time axis of the signal. For this test, the initial sampling schemes have the parameters outlined in Section 4.1. For each type of the initial scheme, we consider 50 realizations. The modified schemes were generated with K=30 for every initial scheme according to the instructions in Section 4.1. We do not change the parameters of the CS reconstruction workflow in order to exclude the parameterization bias and highlight differences due to specific sampling patterns in the sampling schemes.

**Figure 6:**
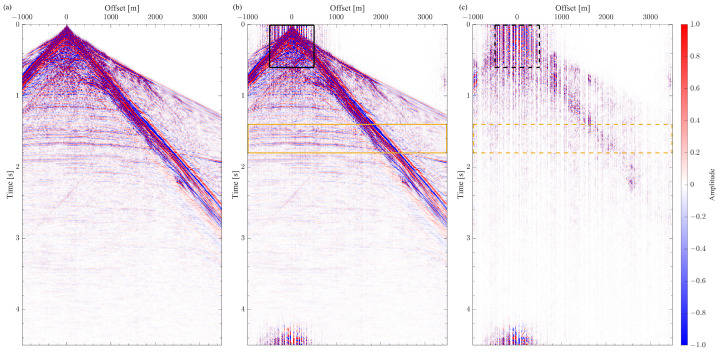
Shot gathers from the CS reconstruction experiment: (**a**) the original shot gather, (**b**) the reconstructed shot gather, (**c**) the difference between the signals in (**a**,**b**). All plots are displayed with the same amplitude range ±1. Strong reconstruction artifacts are present at the near offset and early time outlined in the black rectangle. These artifacts do not contaminate the reflection events outlined in the yellow rectangle.

**Figure 7:**
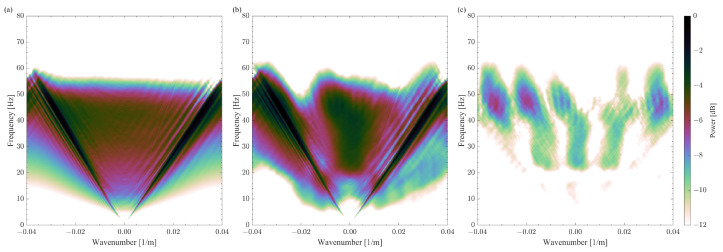
The Fourier spectra of (**a**) the original shot gather, (**b**) the CS reconstructed shot gather, (**c**) the difference between the signals in Figure 6a,b. All plots are displayed with the same power range from −12 to 0 dB. The reference power is the same for all plots.

We consider a shot gather as a seismic signal from the SEAM Barrett model dataset [41]. The shot gather contains 360 receivers spaced by 12.5 m and has a wide range of amplitudes (Figure 6a). Namely, the amplitudes of the surface waves range from −500 to 500, while the amplitudes of the reflected waves are within ±1. None of the following is applied to the signal: amplitude correction, clipping, zero-padding, and smoothing functions at the edges. Also, no noise is added to the synthetic data. The Fourier spectrum of the shot gather shows that a 12.5 m spacing provides the alias-free sampling of the seismic signal (Figure 7a). The curvelet transform is used to obtain a sparse representation of the seismic signal [42]. The SPGL1 solver is used as an algorithm for CS data reconstruction [43]. For each reconstruction experiment, the solver runs until the predefined tolerance level is achieved, leading to a slightly different number of iterations for each reconstruction experiment.

Figure 6b shows an example of the CS-reconstructed signal. In this case, the initial two-step scheme with the recursive gap-filling option decimates the signal prior to the CS reconstruction step. We do not display the reconstruction results obtained with other sampling schemes since they appear to perform very similarly to the one in Figure 6b. The difference between the original and the reconstructed signals shows that the reconstruction algorithm cannot correctly recover all features of the signal (Figure 6c). One can notice the reconstruction noise after 4 s. This noise is related to the wraparound effect of the Fourier transform, which is used in the curvelet to decompose the signal. This effect can be mitigated by extending the signal with zeros along the time axis. This shot gather does not have events of interest after 4 s. Consequently, this wraparound artifact, in this case, is not considered a significant issue.

The CS reconstruction workflow struggles significantly with the portion of the signal outlined in the black rectangle. The seismic shot gather has a huge spike in the amplitude at the very first arrivals for the traces with offsets smaller than 100 m. We make an analogy with a delta function to rationalize the adverse effects of such an amplitude spike on the reconstruction results. It is known that a delta function in the time and space domain would spread over the entire frequency-wavenumber spectrum. In the context of the curvelet transform, which we use as a sparse transform in these experiments, a delta function in the time and space domain will assign rather large values for all curvelet coefficients whose basis functions pass through this location. Some of these strong coefficients will correspond to the basis functions with orientation parallel to the time axis. The proper coefficients of such vertically oriented curvelets are very hard to reconstruct precisely because we decimate the traces in our numerical experiment. The authors in [13] demonstrated that, once the gap in the signal exceeds the size of the curvelet basis function, the corresponding coefficients will not be recovered correctly. Thus, obtaining all coefficients needed to describe the amplitude spike is challenging for the CS reconstruction algorithm. Consequently, the quality of the reconstructing signal around that amplitude spike is also compromised. We may achieve better results by applying, for instance, a median filter. Instead, we performed the CS reconstruction of the seismic signal as it was generated by a seismic wavefield modeling tool without altering the signal in any way.

The Fourier spectra of the reconstructed and difference signals are also affected by the incorrect reconstruction of the curvelet coefficients (Figure 7b,c). Namely, the largest difference in Figure 7c is at the frequency bands of 40–80 Hz and 20–40 Hz. These frequency bands correspond to the scales at which the vertically oriented curvelet basis functions are very thin and may be missed entirely due to the gap size of 3 grid steps. Furthermore, we analyze the CS reconstruction results quantitatively by computing the trace reconstruction error $\kappa_{i}$ as follows:

(5) $$\kappa_{i}=20\log_{10}\frac{∥{\hat{w}}_{i}-w_{i}{∥}_{2}}{∥w_{i}{∥}_{2}},$$

where ${\hat{w}}_{i}$ and $w_{i}$ are the *i*-th traces in the reconstructed and original shot gathers, respectively. The smaller the error, the better the CS reconstruction. We use the time window of 1.4–1.8 s, which mainly contains the reflected events, to compute the trace reconstruction error (the yellow rectangle in Figure 6c).

**Figure 8:**
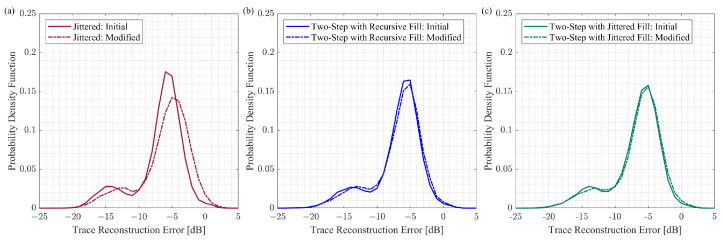
The probability density functions computed based on the CS reconstruction results for the different schemes: (**a**) jittered, (**b**) two-step with the recursive gap-filling option, (**c**) two-step with the jittered gap-filling option. For the modified schemes, the case of K=30 is considered. The trace reconstruction error is computed based on Equation (5).

We build the histograms of the trace reconstruction error based on 50 CS reconstruction trials per group, and each trial has 180 reconstructed traces. We use the kernel distribution for each histogram to obtain a non-parametric representation of the probability density function of a trace reconstruction error. Figure 8 summarizes the probability density functions for the CS reconstruction experiments with initial and modified schemes generated by the different approaches.

**Figure 9:**
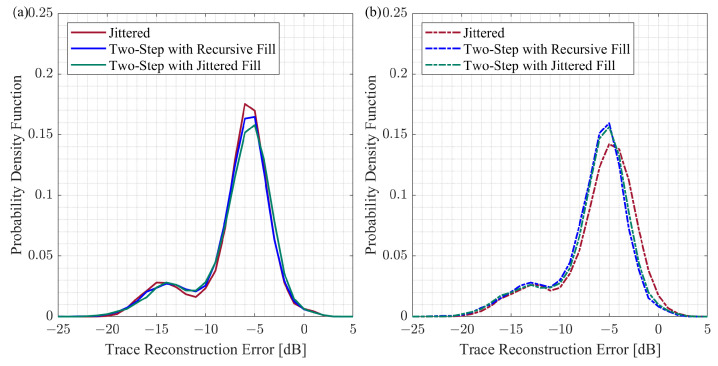
The comparison of the CS reconstruction results for the jittered schemes, the two-step schemes with the recursive gap-filling option, and the two-step schemes with the jittered gap-filling option: (**a**) initial schemes and (**b**) modified schemes with K=30. The trace reconstruction error is computed based on Equation (5).

The probability density functions have two peaks. For the traces that include the surface waves, the trace reconstruction error is within the range from −20 dB to −10 dB. As for the traces that have reflected events, the trace reconstruction error ranges from −10 dB to 0 dB. The probability density functions are very close for the initial and modified two-step sampling schemes (Figure 8b,c). In the case of the jittered approach, the probability density function is shifted to the right for the modified schemes compared to the initial schemes (Figure 8a), implying that the reconstruction results deteriorate for the modified jittered schemes. The comparison between different methods for initial and modified schemes is shown in Figure 9. This comparison reveals that the probability density functions are very similar for all three groups of initial schemes (Figure 9a). However, the probability density function for the modified jittered schemes is shifted to the right compared to the probability density functions of the modified two-step schemes (Figure 9b). It is important to note that, in this experiment, we are considering the change in the reconstruction error due to sampling scheme modification. As for the absolute values of the reconstruction error, these values can change if we consider a different signal or apply another CS reconstruction workflow. Further discussion of the CS reconstruction error is provided in Section 5.

### 4.4. Three-Dimensional Orthogonal Survey Layout

The 3D orthogonal survey layout is considered to analyze the influence of different sampling approaches on the distribution of traces within bins, offsets, and azimuths. The layout has the following parameters: 81 square km covered by 46 source lines orthogonal to 46 receiver lines with 200 m spacing between the lines. Each receiver (or source) line has 360 receivers (or sources), and the distance between adjacent receivers (or sources) is 25 m. The receiver patch covers all 81 square km, which means that each receiver listens to all sources. In addition, the source and receiver lines are shifted by 12.5 m to avoid reciprocal traces.

The full 3D survey is decimated according to the CS-based schemes to build a CS-based survey layout. After decimation, each source (or receiver) line contains only half of the sources (or receivers), which decreases the total number of traces by four compared to the full survey. The CS-based schemes were generated according to the details in Section 4.1. Thus, the CS-based survey layout is evaluated for three groups of initial schemes and three groups of modified schemes with K=30.

**Figure 10:**
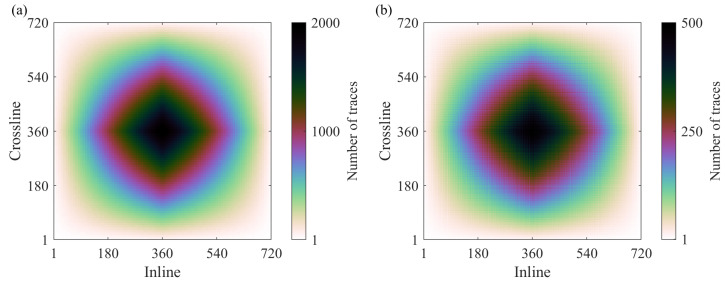
The full fold maps for (**a**) the full and (**b**) the CS-based surveys. The distribution of traces within the fold map for the CS-based survey resembles one for the full survey. However, grid-like features are imprinted into the fold map of the CS-based survey.

**Figure 11:**
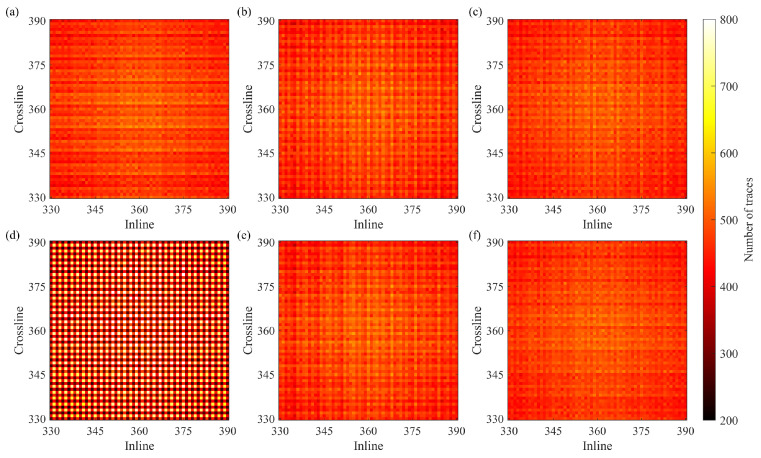
The zoomed portion of the fold maps for the CS-based surveys in the case of (**a**) the initial jittered schemes, (**b**) the initial two-step schemes with the recursive gap-filling option, (**c**) the initial two-step schemes with the jittered gap-filling option, (**d**) the modified jittered schemes, (**e**) the modified two-step schemes with the recursive gap-filling option, (**f**) the modified two-step schemes with the jittered gap-filling option. The fold map for the CS-based survey built based on modified jittered schemes stands out from all other cases because it has large variations in the trace count across the bins.

We calculate the fold maps with 12.5 m by 12.5 m bins for each survey. Figure 10 shows the fold maps of the full survey and the CS-based survey created according to the initial two-step schemes with the recursive gap-filling option. The full fold of the CS-based survey is about four times smaller than the full fold of the full survey, but the distributions of the traces within bins have very similar patterns for both surveys. Figure 11 shows zoomed portions of the fold maps for the CS-based surveys built with the initial and modified schemes. We observe that the reallocated sources and receivers do not change the distributions of the traces in the case of the two-step sampling schemes (Figure 11b,c,e,f). However, the modified jittered schemes lead to a less even distribution of traces within the bins than the distribution of traces achieved by using the initial jittered schemes (Figure 11a,d). In Figure 11d, the bins with more traces regularly alternate those with fewer traces.

**Figure 12:**
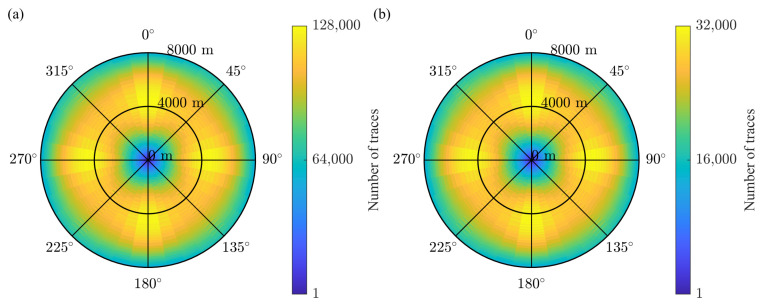
The rose diagrams for (**a**) the full and (**b**) the CS-based surveys. Similar to fold maps in Figure 10, the distribution of trace for different offsets and azimuths have a similar structure for the full and CS-decimated surveys.

**Figure 13:**
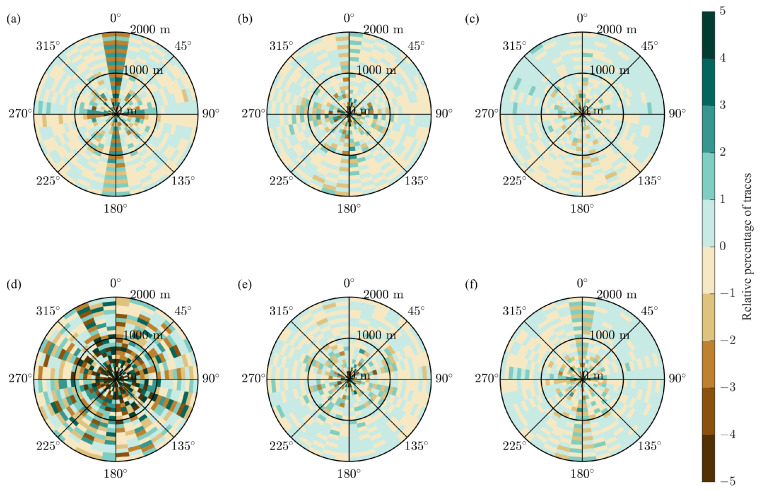
The near offset section of the relative rose diagrams for the CS-based surveys in the case of (**a**) the initial jittered schemes, (**b**) the initial two-step schemes with the recursive gap-filling option, (**c**) the initial two-step schemes with the jittered gap-filling option, (**d**) the modified jittered schemes, (**e**) the modified two-step schemes with the recursive gap-filling option, (**f**) the modified two-step schemes with the jittered gap-filling option. The relative percentage of traces is computed according to Equation (6). For the case of the modified jittered scheme, the values of the relative percentage are the largest compared to the other evaluated cases.

The rose diagrams are computed with bins that have a 10 degree step in azimuth and a 100 m step in offset. Figure 12 shows the rose diagrams for the full survey and the CS-based survey built with the initial two-step schemes with the recursive gap-filling option. Like the fold maps in Figure 10, the rose diagrams are different by the trace count and similar in the trace distribution across the bins. To analyze the subtle changes in the distribution of traces due to the CS-based sampling, we computed the relative rose diagram $\Theta^{rel}$ as

(6) $$\Theta^{rel}=\frac{0.25\Theta^{full}-\Theta^{CS}}{0.25\Theta^{full}}$$

where the factor 0.25=0.5·0.5 is due to the reduction in the number of sources and receivers in each line by the factor of two, the index *i* enumerates the bins in the rose diagram, and $\Theta^{full}$ and $\Theta^{CS}$ are the rose diagrams for the full and CS-based surveys, respectively. We observe that the relative percentage of traces ranges from −2 to 2 for all bins at different azimuths for the initial and modified two-step schemes (Figure 13b,c,e,f). In contrast to that, the initial jittered schemes produce the relative rose diagram in which bins at the azimuths of 0 degrees and 180 degrees have greater variations in the relative percentage of traces than bins at all other azimuths (Figure 13a). As for the modified jittered schemes, the uneven distribution of traces across the relative rose diagram spreads for other azimuth directions (Figure 13d).

## 5. Discussion

In the literature, CS-based seismic acquisition is referred to as sparse, irregular, nonuniform, or randomized acquisition. However, using these terms interchangeably may be confusing because these terms also describe survey layouts not necessarily designed by CS sampling methods. For example, a sparse acquisition may refer to a Nyquist-based layout with coarsely spaced receivers (or sources). As for an irregular acquisition, even a conventional survey may be called irregular due to different obstructions in the field that cause arbitrary deviations in the planned layout. Moreover, regular sampling in one domain (source and receiver coordinates) may be considered irregular or nonuniform in another domain (midpoint coordinates, offset, and azimuth). Therefore, to avoid confusion, we exclusively use the term “CS-based” while referring to sampling schemes built for seismic CS applications.

The current formulation of the two-step sampling method is built based on the CS reconstruction guarantees available in the literature for the Fourier basis. Our formulation can be generalized. For instance, during the first stage, one can use a sampling scheme other than random as long as it satisfies the restricted isometry property. Possible candidates for the first-step scheme can be found in [36]. Such potential generalization of the two-step sampling can help to resolve one of the inconsistencies of the current sampling method. Although the theoretical guarantees we considered are valid for the Fourier basis, the CS reconstruction with such a basis can be poor for complex seismic signals. More suitable basis functions, such as the curvelet transform, do not have known theoretical guarantees on which we can rely to build a sampling method.

In our method, the null space property does not impose any restrictions on the locations of the designated samples. Thus, the second-step samples can be used to incorporate other high-priority constraints on the survey layout. For instance, instead of considering the fixed maximum gap size, one can vary the maximum allowed gap size over the area. This strategy can be effective for surveys with variable near-surface conditions and different levels of background noise caused by external factors.

One may be interested in filling in large gaps in the first-step scheme with a sampling strategy that does not have any element of random sampling. One of the simplest strategies is to place additional samples within large gaps equidistantly. In this case, second-step samples will be separated regularly by small gaps of the same size. Such sampling schemes are not recommended since they are known to contribute to aliasing artifacts that contaminate CS-reconstructed signals. Another possible strategy is to distribute second-stage samples within large gaps based on a certain criterion of sampling scheme quality. In this situation, depending on the implementation and complexity of an objective function, the elements of random sampling can still be involved in finding a set of samples that minimize or maximize the considered criterion.

Lastly, we may consider using the first-stage random scheme itself to fill in large gaps. For instance, if we are interested in reducing the size of the large gap from 20 to 3 grid steps, then the first-stage schemes can be searched for a section of length 20 with a gap not exceeding 3 grid steps, and if this section is found, it can be inserted into the considered gap. If this section is not found, then one needs to come up with further considerations. If the first-stage scheme contains several sections of length 20 with a gap not exceeding 3 grid steps, then the choice becomes non-unique. Consequently, unless specific preferences are imposed on the choice, one section can be chosen uniformly at random from the other detected sections with the same length and maximum gap size. This strategy can be intriguing because it can make the first-stage schemes self-sufficient for gap-filling purposes, which in turn reduces the reliance on supplemental sampling strategies for the second step.

We propose our sampling method as a two-step design process, which implies that random and designated samples are not distinguished during the executing stage of the survey project. Yet, we provide here our reasoning for the circumstances when the separation between random and designated samples during the field implementation can be beneficial. For example, in simultaneous source acquisition, seismic vibrators that shoot at random locations may be separated by a time delay (several hours) from the seismic vibrators that shoot at locations strategically chosen based on previous random samples. This scenario may be beneficial when the surface terrain and obstacles disturb a typical time and offset separation scheme required to ease the deblending process.

The overview section highlights the differences between several CS-based sampling methods. At the same time, all methods we consider in this paper have one common characteristic. They do not impose or require prior knowledge of the subsurface either in the form of a velocity model or recorded wavefield over the area. These methods can be in demand when the details of the velocity model or legacy data are limited, e.g., exploration surveys. Once more information about subsurface geology is available, model-based methods and machine learning can be applied to take advantage of this information for survey design [44,45]. These methods can be valuable for designing monitoring time-lapse surveys. It is not excluded that different sampling methods will be used over the same survey area in case there will be multiple survey campaigns. Consequently, it may be worth comparing and understanding the differences between sampling schemes generated by general sampling methods and ones that utilize and rely on prior information about the subsurface.

We analyzed the average Fourier spectra and showed that different sampling schemes have different noise patterns in the wavenumber domain. The presence of the spike at the normalized wavenumber of 0.5 may indeed negatively affect the CS reconstruction results since such a spike indicates the presence of aliasing in the sampling scheme. Although we do not observe a strong relationship between the shape average Fourier spectra and the CS reconstruction results, the role of the average Fourier spectra of the sampling scheme on other processing steps may be interesting to investigate.

We used the trace reconstruction error to assess the CS reconstruction results qualitatively with different sampling schemes. Even though this measure is often used in the CS literature, its usefulness for seismic CS application is uncertain. Firstly, measuring reconstruction errors in the shot or receiver gather domains is not straightforwardly translated into the final products of the seismic interpretation and analysis. The reconstructed gathers will go through a comprehensive processing workflow with a number of steps. The choice of the parameters for the processing steps depends on both data quality and processor decision-making. Consequently, it may be hard to predict how the features of the reconstruction noise are mapped into the final results.

The direct comparison of reconstruction error values reported in different publications should be conducted carefully, considering various factors. One factor contributing to the reconstruction error is the sparsity level (Equation (A7)). Sparsity levels of the seismic signals are not quantified in many publications. Thus, one can only make qualitative assumptions on the sparsity level based on the visual appearance of the seismic signal. Reprocessing of the seismic gathers is another factor that can challenge the comparison of the reconstruction error values. The prepossessing of the data can be conducted with slightly different parameters, and such procedures are not always reported in an explicit manner.

In this paper, we restricted our reconstruction experiments to a commonly used CS-reconstruction workflow, which uses the curvelet transform and the basis pursuit algorithm (the solver). Such a choice was made since, for this interpolation workflow, the interaction of the user with the solver is minimal, which helps to remove the parameterization bias of a specific user and reveal differences associated with specific sampling patterns. Many other interpolation techniques available in the literature can potentially be explored, some of which can be less sensitive to specific sampling patterns. One of the commonly used seismic data interpolation techniques is the 5D interpolation method [46]. This technique has been found to be effective for preparing the data for subsequent seismic data processing steps such as migration. For this purpose, the input signal of the 5D interpolation workflow is a denoised common midpoint gather. In other words, linear noise, such as surface wave energy, will be suppressed before interpolation. As for our reconstruction experiments, the workflow was able to handle the recorded wavefield in the shot gather domain, which includes the surface wave energy.

We studied the application of CS-based sampling for seismic surveys with the orthogonal layout. In our examples, we considered decimating source (or receiver) stations within the source (or receiver) lines along the densely sampled direction. Decimating the survey lines may not be favorable for CS reconstruction because the seismic wavefield may be sampled too coarsely in the direction perpendicular to the lines. The two-step sampling may be applied to the other survey layouts. It is worth noting that CS-based surveys may not be necessarily built by decimating the regular layout. In CS, the number of successfully reconstructed samples is constrained by the sparsity level of a signal and the number of active measurements.

## 6. Conclusions

A novel two-step sampling method is introduced for designing a CS-based layout for seismic applications. This method uses a random sampling scheme during the first step, and then designated samples are added to comply with the seismic survey requirements and constraints. From the CS theory point of view, unlike the other methods, the new method is supported by the restricted isometry property and the null space property. Furthermore, as opposed to the commonly used CS-based methods, the two-step sampling method is flexible and can be applied in the presence of large obstructions in the field, and it does not limit the choice of seismic survey parameters.

The conducted numerical experiments provide a comprehensive examination of the two-step schemes. The CS reconstruction tests demonstrate that two-step schemes have comparable CS reconstruction performance with the initial jittered schemes. Also, the relative rose diagrams show that the two-step sampling provides a more uniform distribution of traces within offset–azimuth bins than the jittered sampling. Finally, comparing the results of numerical experiments for initial and modified two-step schemes, we observe that the average Fourier spectra, the probability density function of the trace reconstruction error, and the distributions of traces in fold maps and rose diagrams are not affected by the reallocated samples. We conclude that two-step schemes are suitable for the implementation of CS-based surveys in the field, which implies that requirements on the prepared locations of source and receiver may be relaxed for the survey crew.

## Data Availability

The seismic shot gather used in the CS reconstruction experiments is from the SEAM Barrett dataset. The latter can be purchased from the SEG Advanced Modeling Corporation (SEAM) website https://seg.org/seam/data-sets/.

## Abbreviations

The following abbreviations are used in this manuscript:

CS	Compressive Sensing
NSP	Null Space Property
RCP	Restricted Isometry property
BPDN	Basis Pursuit Denoising
RIC	Restricted Isometry Constant
SEAM	SEG Advanced Modeling
SEG	Society of Exploration Geophysicists
SPGL1	Spectral Gradient-Projection Method for Minimization L1 Norm

## Appendix A. Compressive Sensing Theoretical Background

Compressive sensing operates with sparse and compressible signals, incomplete measurements, and non-linear reconstruction algorithms. This section provides a short mathematical introduction to the CS framework in light of its seismic application. The concept of the signal sparsity is at the core of the CS framework. An *s*-sparse signal is a signal with at most *s* nonzero entries:

(A1) $$∥\hat{x}{∥}_{0}:=\mathrm{card}\left(\left\{l\in \left[L\right]:{\hat{x}}_{l}\neq 0\right\}\right)\leq s.$$

The operator ${∥\cdot ∥}_{0}$ counts nonzero entries of the vector $\hat{x}\in C^{L}$ and the operator card denotes the cardinality of a set. Equation (A1) describes perfectly sparse signals, which may be an overly idealized mathematical representation of real-world signals. The concept of signal compressibility uses the error of the best *s*-term approximation to address signals with sparsity defects. The error of the best *s*-term approximation measures the closeness of a compressible signal $x\in C^{L}$ to a perfectly *s*-sparse signal $\hat{x}\in C^{L}$:

(A2) $$\sigma_{s}{\left(x\right)}_{p}=\inf\{∥x-\hat{x}{∥}_{p},\hat{x}\in C^{L}\;\mathrm{is}\;s\text{-}\mathrm{sparse}\},$$

where p>0 and the operator inf takes the infimum of a function. The signal x is compressible when the error of its best *s*-term approximation decays rapidly in *s* [12].

The seismic wavefield is not inherently sparse but can be transformed into a compressible signal. Namely, the wavefield $w\in R^{N}$ and its compressible version $x\in C^{L}$ are connected by the sparse transform matrix $C\in C^{L\times N}$

(A3) $$x=\mathrm{Cw}\;\mathrm{and}\;w=C^{H}x,$$

where *H* is the Hermitian transpose. The transform matrix C can be an orthonormal basis (e.g., the Fourier transform, N=L) or a tight frame (e.g., the curvelet transform, 7N≈L). The choice of the transform depends on the complexity of the wavefield and the sorting of the seismic data volume. A detailed review of other useful sparse transforms with localized basis functions (e.g., seislet, shearlet, coutrolets, etc.) and evaluation of two-step sampling performance using these sparse transforms are out of the scope of this paper.

Another essential element of the CS framework is the sensing matrix $A\in C^{M\times L}$ (M<L), which connects the sparse transform matrix and a CS-based scheme as follows:

(A4) $$A=RC^{H}.$$

The matrix $R\in R^{M\times N}$ (M<N) is a binary matrix with a single one on each row. The position of a nonzero element within each row is defined according to the CS-based scheme. Thus, compressive sensing of a signal is equivalent to its simultaneous recording and compression:

(A5) $$d=Rw=RC^{H}x=\mathrm{Ax},$$

where $d\in R^{M}$ is the acquired seismic wavefield. In CS seismic applications, the decimation ratio $\frac{M}{N}$ typically ranges from 0.25 to 0.75 [47]. However, the decimation ratio should be balanced with the level of noise in the data [48].

The last essential element of CS is the reconstruction algorithm, which solves the underdetermined system of linear equations (Equation (A5)) to obtain the seismic wavefield in the omitted samples based on the recorded data. One example of the reconstruction algorithm is basis pursuit denoising (BPDN):

(A6) $$z=\arg\min_{z^{\prime }\in C^{L}}∥z^{\prime }{∥}_{1}\;\;\mathrm{subject}\;\mathrm{to}\;\;{∥d-Az^{\prime }∥}_{2}\leq e,$$

where *e* is the noise level. Equation (A6) uses the $\ell_{1}$ norm to measure the sparsity of the signal, while in the definition of the *s*-sparse signal, the nonzero elements of the signal are counted to determine the sparsity level (Equation (A1)). This discrepancy is caused by the intractability of the ideal recovery scheme — the problem in Equation (A6) with the operator ${∥\cdot ∥}_{0}$ instead of the $\ell_{1}$ norm. Measuring the sparsity of signals by means of the $\ell_{1}$ norm makes the program in Equation (A6) not only tractable but also convex. In addition, the $\ell_{1}$ norm is a valid replacement for the operator ${∥\cdot ∥}_{0}$ under certain conditions [12].

The reconstructed signal z, a solution of BPDN, approximates the unknown compressible signal x as

(A7) $${∥x-z∥}_{2}\leq c_{1}s^{-\frac{1}{2}}\sigma_{s}{\left(x\right)}_{p}+c_{2}e,$$

where $c_{1}>0$ and $c_{2}>0$ are constants that depend on the properties of the sensing matrix A. A sensing matrix with small $c_{1}$ and $c_{2}$ may not necessarily promote the reconstruction of the signal z closest to the unknown signal x compared to other signals reconstructed from the measurements made by means of other sensing matrices. On the one hand, small $c_{1}$ and $c_{2}$ lead to a small theoretical reconstruction error (the right-hand side of Equation (A7)), and the corresponding sensing matrix may be favorable for measurements. On the other hand, the right-hand side of Equation (A7) provides only an upper bound on the empirical reconstruction error (the left-hand side of Equation (A7)), which means that this error may not be strictly controlled by the constants $c_{1}$ and $c_{2}$. The empirical reconstruction error may be controlled and predicted when a signal model is used to build a sensing matrix, e.g., in the case of time-lapse seismic.

## Appendix B. Tools for Analyzing CS-Based Sampling Schemes

The sensing matrix A should be specially constructed to meet the requirements of the seismic survey and enable data reconstruction for omitted sources and receivers. The CS framework uses metrics and matrix properties to analyze the goodness of the sensing matrix for the data reconstruction process. For example, the $\ell_{2}$ robust null space property (NSP) may be used to verify whether the sensing matrix is suitable for the reconstruction of approximately sparse signals from noisy measurements. The authors of [12] define this property as follows: the matrix $A\in C^{M\times L}$ is said to satisfy the $\ell_{2}$-robust NSP of order *s* (with respect to ∥·∥) with constants 0<ρ<1 and τ>0 if, for any set S⊂[L] with card(S)≤s,

(A8) $$∥v_{S}{∥}_{2}\leq \rho s^{-\frac{1}{2}}∥v_{\bar{S}}{∥}_{1}+\tau ∥\mathrm{Av}∥\;\;\mathrm{for}\;\mathrm{all}\;\;v\in C^{L}.$$

The $\ell_{2}$ robust NSP is not computationally tractable and thus not very applicable for CS-based scheme design. Another property, the restricted isometry property (RIP), is also computationally intractable. However, different classes of randomly constructed matrices satisfy the RIP with high probability. According to [12], a matrix $A\in C^{M\times L}$ is said to satisfy the RIP of order *s* if there exists 0<δ<1 such that

(A9) $${\left(1-\delta \right)∥x∥}_{2}^{2}\leq {∥\mathrm{Ax}∥}_{2}^{2}\leq \left(1+\delta \right){∥x∥}_{2}^{2},$$

for all *s*-sparse vectors $x\in C^{L}$. The smallest δ that satisfies the above inequality is called the restricted isometry constant (RIC) and is denoted as $\delta_{s}=\delta_{s}\left(A\right)$.

A more popular metric to assess the goodness of the CS-based scheme is the coherence due to its computability. According to [12], the coherence μ=μ(A) of the matrix A with columns $a_{1},a_{2},\ldots a_{L}$ is defined as

(A10) $$\mu =\max_{1\leq k\neq l\leq L}\frac{\left|\langle a_{k},a_{l}\rangle \right|}{∥a_{k}{∥}_{2}{∥a_{l}∥}_{2}}.$$

The coherence μ is bounded from above by one and from below by the Welch bound $\mu_{W}$ as follows:

(A11) $$\mu_{W}=\sqrt{\frac{L-M}{M(L-1)}}\leq \mu \leq 1.$$

In most cases, the coherence is computed when the Fourier basis is considered for the sparse transform C. In this scenario, the coherence is equal to the maximum non-DC amplitude of the Fourier spectrum of the sampling scheme. From the CS theoretical point of view, to reconstruct the *s*-sparse signal, it is sufficient that the coherence satisfies the following inequality:

(A12) $$\mu <\frac{1}{2s-1}.$$

In practice, this inequality is rarely satisfied. For example, suppose the coherence value of a seismic CS-based scheme is 0.1. Then, the seismic volume in the transform domain should have only 5 non-zero coefficients, which is a very unrealistic scenario.

The Fourier spectrum of a sampling scheme is yet another tool to analyze the properties of CS-based schemes. Although the CS theoretical guarantees are available only for the maximum non-DC amplitude of the Fourier spectrum of sampling schemes, in practice, the analysis of such spectrum is commonly used in CS-based scheme design [18].

In a seismic survey, sources are coupled with receivers because each active receiver records the seismic wavefield generated by each active source. Furthermore, a unique source–receiver pair produces a trace in the recorded data volume. The distribution of traces within offsets, azimuths, and bins can be analyzed by means of attributes from seismic survey design. For example, a fold map splits a survey area into bins, showing how many traces belong to the same bin. Another example is a rose diagram, which displays the number of traces for each offset–azimuth bin. A detailed description of how to compute the fold maps, rose diagrams, and other attributes can be found in books on seismic acquisition design [49].

## Appendix C. The $\ell_{2}$-Robust NSP and Additional Rows in the Sensing Matrix

Let us build a new matrix $\hat{A}\in C^{M_{A}\times L}$ by appending new rows into the matrix $A\in C^{M_{R}\times L}$:

(A13) $$\hat{A}:=\left[\frac{A}{B}\right],$$

where the matrix $B\in C^{M_{D}\times L}$ includes the new additional rows and $M_{R}+M_{D}=M_{A}<L$. For any vector $v\in C^{L}$, the following inequality is true:

(A14) $$∥Av∥\;\leq \;∥\hat{A}v∥,$$

because the vector $∥\hat{A}v∥$ contains all elements of the vector ∥Av∥ and additional elements due to the matrix B. For example, if ∥·∥ is the $\ell_{2}$ norm, then Equation (A14) is equivalent to

(A15) $$\sqrt{\sum_{i=1}^{M_{R}}(\langle a_{i}^{H},v\rangle }{)}^{2}\leq \sqrt{\sum_{i=1}^{M_{R}}{\left(\langle a_{i}^{H},v\rangle \right)}^{2}+\sum_{i=1}^{M_{D}}{\left(\langle b_{i}^{H},v\rangle \right)}^{2}},$$

where $a_{i}$ and $b_{i}$ are the rows of the matrices A and B, respectively. Furthermore, we consider that the matrix $A\in C^{M_{R}\times L}$ ($M_{R}<L$) has the $\ell_{2}$-robust NSP of order *s* with constants 0<ρ<1 and τ>0. In other words, the following inequality holds:

(A16) $$∥v_{S}{∥}_{2}\;\leq \;\rho s^{-\frac{1}{2}}∥v_{\bar{S}}{∥}_{1}+\;\tau ∥\mathrm{Av}∥$$

for any set S⊂[L] with card(S)≤s and $v\in C^{L}$. Combining Equations (A14) and (A16), we deduce that

(A17) $$∥v_{S}{∥}_{2}\;\leq \;\rho s^{-\frac{1}{2}}∥v_{\bar{S}}{∥}_{1}+\;\tau ∥\hat{A}v∥.$$

Thereby, the new matrix $\hat{A}\in C^{M_{A}\times L}$ has the $\ell_{2}$-robust NSP of order *s* with constants 0<ρ<1 and τ>0 for all $v\in C^{L}$ and any set S⊂[L] with cardinality *s*.
